# Artificial Intelligence in Colon Capsule Endoscopy—A Systematic Review

**DOI:** 10.3390/diagnostics12081994

**Published:** 2022-08-17

**Authors:** Sarah Moen, Fanny E. R. Vuik, Ernst J. Kuipers, Manon C. W. Spaander

**Affiliations:** Department of Gastroenterology and Hepatology, Erasmus MC University Medical Center, 3015 CE Rotterdam, The Netherlands

**Keywords:** colon capsule endoscopy, artificial intelligence, polyp detection, bowel cleansing

## Abstract

**Background and aims**: The applicability of colon capsule endoscopy in daily practice is limited by the accompanying labor-intensive reviewing time and the risk of inter-observer variability. Automated reviewing of colon capsule endoscopy images using artificial intelligence could be timesaving while providing an objective and reproducible outcome. This systematic review aims to provide an overview of the available literature on artificial intelligence for reviewing colonic mucosa by colon capsule endoscopy and to assess the necessary action points for its use in clinical practice. **Methods**: A systematic literature search of literature published up to January 2022 was conducted using Embase, Web of Science, OVID MEDLINE and Cochrane CENTRAL. Studies reporting on the use of artificial intelligence to review second-generation colon capsule endoscopy colonic images were included. **Results**: 1017 studies were evaluated for eligibility, of which nine were included. Two studies reported on computed bowel cleansing assessment, five studies reported on computed polyp or colorectal neoplasia detection and two studies reported on other implications. Overall, the sensitivity of the proposed artificial intelligence models were 86.5–95.5% for bowel cleansing and 47.4–98.1% for the detection of polyps and colorectal neoplasia. Two studies performed per-lesion analysis, in addition to per-frame analysis, which improved the sensitivity of polyp or colorectal neoplasia detection to 81.3–98.1%. By applying a convolutional neural network, the highest sensitivity of 98.1% for polyp detection was found. **Conclusion**: The use of artificial intelligence for reviewing second-generation colon capsule endoscopy images is promising. The highest sensitivity of 98.1% for polyp detection was achieved by deep learning with a convolutional neural network. Convolutional neural network algorithms should be optimized and tested with more data, possibly requiring the set-up of a large international colon capsule endoscopy database. Finally, the accuracy of the optimized convolutional neural network models need to be confirmed in a prospective setting.

## 1. Introduction

Colon Capsule Endoscopy (CCE) provides a promising non-invasive alternative to colonoscopy for exploration of the colonic mucosa [1,2]. It uses an ingestible, wireless, disposable capsule to explore the colon without the need for sedation or gas insufflation. The first generation CCE was introduced in 2006 and a second generation CCE was developed in 2009 (PillCam Colon 2, Medtronic, Minneapolis, MN, USA) [3]. The second generation colon capsule endoscopy (CCE-2) has a high diagnostic accuracy for the detection of colorectal polyps, with a sensitivity of 85% and specificity of 85% for polyps of any size, sensitivity of 87% and specificity of 88% for polyps ≥ 6mm, and a sensitivity of 87% and specificity of 95% for polyps ≥ 10 mm [4]. 

An important limitation of the applicability of CCE in daily practice is the accompanying labor-intensive reviewing time for the CCE images. A recent study showed a median reading time of 70 min for the entire gastrointestinal tract and 55 min for review of the colon alone [5]. On top of that, agreement in and between different readers may also be a topic of concern. Literature regarding intra- and inter-observer variability in reviewing CCE images is scarce, but one study demonstrated a poor level of agreement among both expert and beginner readers in determining the indication for follow-up colonoscopy based on the number and size of detected polyps [6]. There was also a poor level of agreement in determining the bowel cleansing quality. 

Automated reviewing of CCE images using artificial intelligence (AI) could be timesaving for clinicians while providing an objective and reproducible outcome. AI is a very broad term that describes a computerized approach that includes machine and deep learning methods for interpreting data that normally requires human intelligence [7,8]. Basic AI methods can classify images by computing scores based on features such as texture and color. Machine learning based on pre-defined features is a another AI method used to classify images, where a classifying algorithm is created based on feature classification by experts. An important example of this method is the support vector machine (SVM). Deep learning is a sub-class of machine learning where features do not have to be pre-defined. It is based on a neural network structure that can learn discriminative features from data automatically, giving them the ability to solve very complex problems. Convolutional neural network (CNN) is the most common deep learning algorithm for classifying images. It uses many images to develop and train a classification model by learning rich features and repeating patterns from these images [9]. 

In colonoscopy, research investigating the use of AI as an aid for the detection of colorectal lesions is already rapidly evolving [10,11]. However, blindly applying the same automated methods to CCE would be blunt due to the differences in the images provided by CCE and colonoscopy. For example, localizing polyps and determining their exact number is more difficult using CCE since the capsule spins around and moves back and forth while the lack of air insufflation causes the intestinal wall to protrude into the lumen, sometimes mimicking polyps. Therefore, a reliable AI method specifically developed for reviewing CCE images is warranted. Some literature is available regarding automated methods for reviewing small bowel capsule endoscopy (SBCE) [7], but literature on AI using CCE is scarce. This systematic review aims to give an overview of the available literature on AI methods for reviewing the colonic mucosa by CCE and assess the necessary action points to evolve AI technology for the use of CCE in daily clinical practice.

## 2. Methods

A systematic search aiming to retrieve published trials and abstracts reporting on AI using CCE was conducted following the guidelines of the Preferred Reporting Items for Systematic Review and Meta-Analyses (PRISMA) [12]. A systematic search was conducted on literature databases from inception until the 4th of January 2022. Embase, Web of Science, OVID MEDLINE and Cochrane CENTRAL were used as potential sources. The search was conducted using controlled vocabulary supplemented with several key words (Table 1).

In 2006, the first-generation colon capsule (CCE-1) was developed, and in 2011, the second-generation colon capsule (CCE-2) came to the market. New technology was implemented in the second-generation colon capsule: the capsule frame rate increased from 4 to 35 images per second; the angle of view increased from 156° to 172° for each lens and the data recorder was improved. The CCE-2 achieved a substantially higher sensitivity and specificity to detect polyps compared to the first-generation colon capsule [3]. Therefore, studies using CCE-1 were excluded. Two independent reviewers (S.M. and F.E.R.V.) first screened the selected studies by title and abstract. Studies reporting on AI for reviewing CCE-2 colonic images were selected. Included studies could report on the use of AI for the detection of abnormalities, determining the location of the capsule in the colon and assessing bowel cleansing quality. A full-text examination of the selected publications was performed by the same reviewers independently. Reference lists of the included studies were hand-searched to identify potentially relevant studies that were not retrieved in the original search. 

Details regarding the development of the proposed AI models and numbers on the performance of these models were extracted from the final set of included studies. A meta-analysis could not be performed due to the heterogeneity of the study designs. 

### Quality Assessment of the Included Studies 

The quality of the included studies in terms of risk of bias and concerns regarding applicability were independently assessed by two reviewers (S.M. and F.E.R.V.) using the Quality Assessment of Diagnostic Accuracy Studies (QUADAS) -2 assessment tool [13]. 

## 3. Results

### 3.1. Literature Search

**Figure 1:**
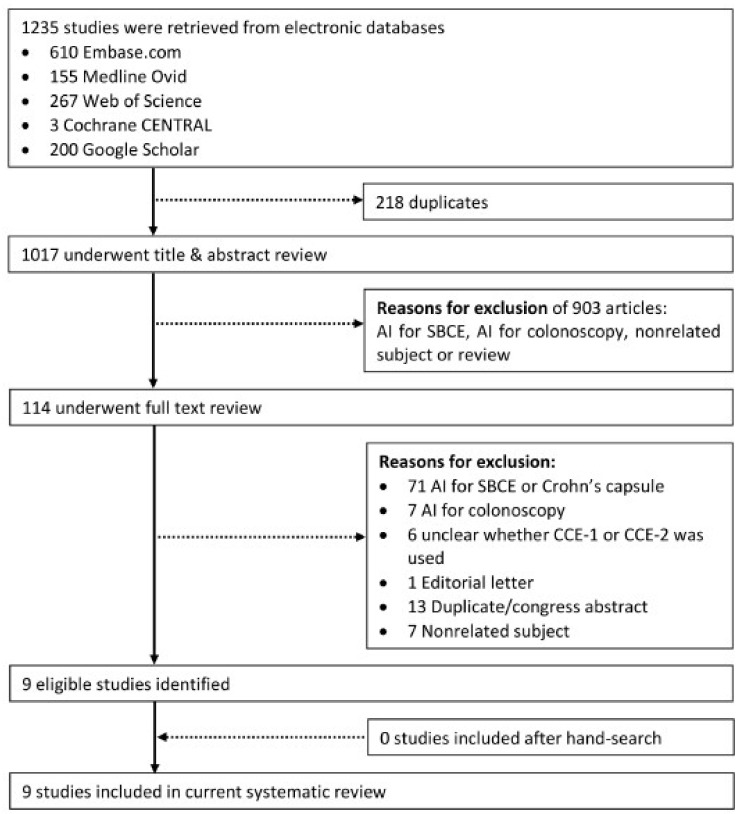
Flow chart of study selection.

After removal of duplicates, retrieved articles were screened for eligibility based on their title and/or abstract (Figure 1). A total of 1017 articles were evaluated for eligibility, after which 903 were excluded. The remaining 114 studies underwent full-text review, after which 105 were excluded for various reasons. No additional studies were retrieved by hand-search. A total of nine studies were included in the final review. 

### 3.2. Study Characteristics

Baseline characteristics of the included studies are shown in Table 2. All included studies were full-text papers presenting cohort studies reporting on AI for reviewing CCE-2 colonic images. Two studies reported on computed assessment of bowel cleansing, five studies reported on computed polyp or colorectal neoplasia detection, one study reported on computed blood detection and one study reported on computed capsule localization. For the studies reporting on bowel cleansing, one study evaluated bowel cleansing for each video frame while the other study evaluated bowel cleansing for the entire video. All other studies evaluated the presence of polyps, presence of blood or capsule localization for each frame. Regarding the AI method, five studies developed a SVM or CNN model, where a selection of frames is needed for the training of the model. To evaluate the performance of the proposed AI methods, all studies used a separate evaluation of the CCE images as a reference. Seven studies used the evaluation of CCE readers as a reference, one study used the known outcomes from a CCE database and one study used the findings from subsequent colonoscopy. 

### 3.3. Quality of the Included Studies

The risk of bias and applicability concerns in the included studies, which were determined by using the QUADAS-2 tool, are presented in Table 3. All studies had a high risk of bias regarding patient selection, since they included CCE videos derived from previous trials or databases and information on the patient population behind the CCE videos was limited or lacking. One study regarding AI bowel cleansing assessment also raised applicability concerns regarding patient selection, since CCE videos were excluded when they were too poor in quality after the first lecture or when the CCE videos were incomplete [14]. Two studies had a high risk of bias regarding their index test, since they determined their models’ optimal cut-off values yielding the highest diagnostic performance by using a ROC curve, which could have led to overoptimistic results, which could likely be poorer when using the same threshold in an independent sample [14,15]. Three studies raised applicability concerns regarding their index test, since they did not report on the performance of their AI models in terms of sensitivity and specificity [16,17,18].

**Table 2:** Characteristics of the nine included studies.

First Author, Year of Publication, Country	Application	Type of AI Method	Evaluation for Each Frame or for Each Video	Included Videos, *n*	Frames Available from These Videos	Frames Available for Training the Model if Applicable	Selected Frames for Testing the Developed AI Method	Reference Group
Becq 2018 France [14]	Bowel cleansing assessment	1. Red over green (R/G ratio) 2. Red over brown (R/(R + G ratio)	Frame	12	79,497	N/A	216 (R/G set) 192 (R/(R + G) set)	2 CCE readers
Buijs 2018 Denmark [16]	Bowel cleansing assessment	1. Non- linear index model 2. SVM model	Video	41	Unknown	Unknown	N/A	4 CCE readers
Figueiredo 2011 Portugal [17]	Polyp detection	Protrusion based algorithm	Frame	5	Unknown	N/A	1700	Subsequent colonoscopy
Mamonov 2014 USA [15]	Polyp detection	Binary classification after pre-selection	Frame	5	18,968	N/A	18,968	Known reviewed CCE dataset
Nadimi 2020 Denmark [19]	Polyp detection	CNN	Frame	255	11,300	7910	1695	Unknown amount of CCE readers
Yamada 2020 Japan [20]	Colorectal neoplasia detection	CNN	Frame	184	20,717	15,933	4784	3 CCE readers
Saraiva 2021 Portugal [21]	Protruding lesion detection	CNN	Frame	24	1,017,472	2912	728	2 CCE readers
Saraiva 2021 Portugal [22]	Blood detection	CNN	Frame	24	3,387,259	4660	1165	2 CCE readers
Herp 2021 Denmark [18]	Capsule localization	T-T model	Frame	84	Unknown	N/A	Unknown	Unknown amount of CCE readers

AI = Artificial Intelligence, SVM = Support Vector Machine; CNN = Convolutional Neural Network, CCE = Colon Capsule Endoscopy, N/A = Not Applicable, R/G = Red over Green, R/(R + G) = Red over Brown.

**Table 3:** QUADAS-2 (Quality Assessment of Diagnostic Accuracy Studies) analysis for the assessment of the risk of bias in the included studies.

	Risk of Bias				Applicability Concerns		
	Patient Selection	Index Test	Reference Standard	Flow and Timing	Patient Selection	Index Test	Reference Standard
Becq [14]	+	+	−	−	+	−	−
Buijs [16]	+	−	−	−	−	+	−
Figueiredo [17]	+	−	−	−	−	+	−
Mamonov [15]	+	+	−	−	−	−	−
Nadimi [19]	+	−	−	−	−	−	−
Yamada [20]	+	−	−	−	−	−	−
Saraiva [21]	+	−	−	−	−	−	−
Saraiva [22]	+	−	−	−	−	−	−
Herp [18]	+	−	−	−	−	+	−

− = low risk of bias; + = high risk of bias.

### 3.4. Artificial Intelligence for the Assessment of Bowel Cleansing Quality in CCE-2

**Figure 2:**
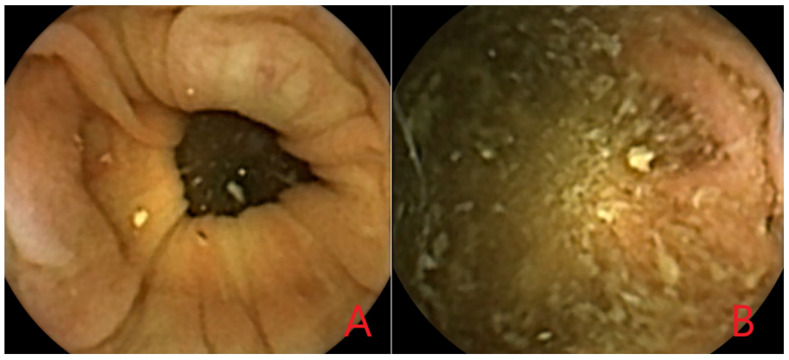
(**A**) Adequately cleansed CCE frame; (**B**) Inadequately cleansed CCE frame.

Two studies reported on computed assessment of bowel cleansing in CCE-2 (Table 4, Figure 2). 

#### 3.4.1. Development of the Proposed AI Models for Computed Assessment of Bowel Cleansing

The first study created two computed assessment of cleansing (CAC) scores using the ratio of color intensities red over green (R/G ratio) and red over brown (R/(R + G) ratio) [14]. After sorting and random selection, for each ratio a set of frames representative of the range of these ratios were obtained. These sets of frames were also evaluated by two experienced CCE readers who were blinded to the CAC scores. The experienced readers classified the frames as having either poor, fair, good or excellent bowel cleansing. Frames with poor or fair quality were defined as inadequately cleansed and frames with good or excellent quality were defined as adequately cleansed. Using the assessment of the experienced reviewers as a reference, the optimal cut-off values yielding the highest diagnostic performance for cleansing assessment were determined for both ratios using a receiver operating characteristic (ROC) curve. 

The second study developed two CAC models, a non-linear index model and a support vector machine (SVM) model [16]. In both models, each pixel was defined as being clean or dirty, after which, the cleanliness of each frame was determined based on the number of clean and dirty pixels it contained. The cleansing level of the complete video was determined by the median cleansing of all frames and weighted based on the number of pixels in the frames. The non-linear index model classified pixels as either clean or dirty based on the distribution of the colors red, green and blue. The SVM model is based on machine learning concepts. A medical doctor classified pixels as being either clean or dirty. Using these evaluated pixels, a SVM algorithm was created through machine learning to assess the cleanliness of each pixel. For defining the cleanliness of each frame, and subsequently for each video, thresholds for unacceptable, poor, fair and good cleansing were predicted and corrected using learning techniques within the algorithm. To be able to evaluate both models, the bowel cleansing quality of each video was also classified by four CCE readers, including two international experts and two medical doctors with short formal training.

#### 3.4.2. Performance of the Proposed AI Models for Computed Assessment of Bowel Cleansing

The CAC scores developed in the first study resulted in a bowel cleansing evaluation for each CCE frame defined as either adequately or inadequately cleansed [14]. The R/G ratio discriminated adequately cleansed frames from inadequately cleansed frames with a sensitivity of 86.5% and a specificity of 78.2%, whereas the R/(R + G) ratio did this with a higher sensitivity of 95.5% but a lower specificity of 63.0%. 

The CAC models developed in the second study resulted in a bowel cleansing classification for each CCE video defined as either unacceptable, poor, fair or good [16]. Evaluation of the performance of their models was not expressed in terms of sensitivity and specificity, but in levels of agreement with the CCE readers. The non-linear index model classified 32% of the videos in agreement with the CCE readers, while the SVM model reached a higher agreement level of 47%. The non-linear index model misclassified 32% of the videos with more than one level of cleanliness compared to 12% in the SVM model. 

### 3.5. Artificial Intelligence for Polyp Detection in CCE-2

**Figure 3:**
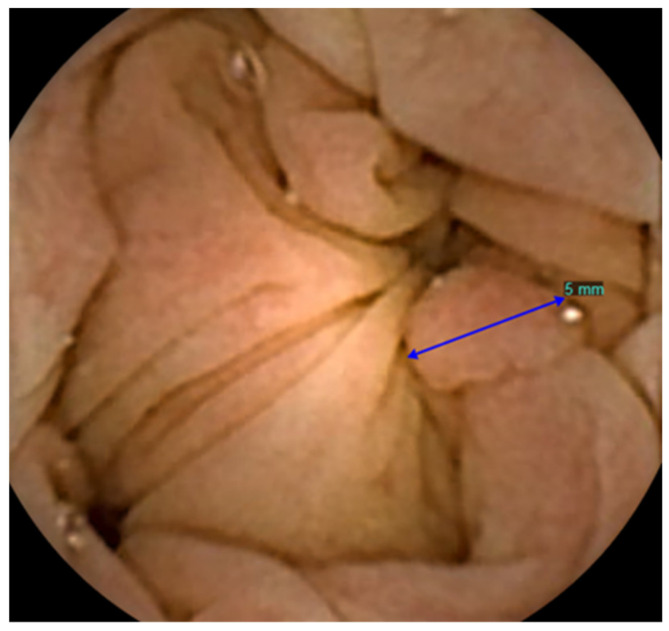
Polyp visualized in CCE.

Five studies reported on AI polyp detection in CCE-2 (Table 5, Figure 3). 

#### 3.5.1. Development of the Proposed AI Models for Polyp Detection

The first two studies developed algorithms for automated polyp detection based on the geometric characteristic of polyps that they have a roundish protrusion into the colonic lumen compared to the surrounding mucosal surface. In the first study, the amount of protrusion was gauged into a special function called P, where the value of P is closely related to the size of the protrusion in the images [17]. Findings from a subsequent colonoscopy were used as a reference to determine which frames contained polyps. In the second study, a binary classification algorithm was developed that resulted in the output “polyp” or “normal” [15]. Frames that potentially contained polyps were first pre-selected based on the texture content. The surface of polyps is often highly textured, however too much texture implies the presence of bubbles or trash liquid. Therefore, in the preselection procedure all frames with too little or too much texture were discarded. Subsequently, a measure of protrusion was created which was used as the decision parameter of the final binary classifier with pre-selection. From the used CCE dataset, it was known which frames contained polyps. Based on the entire dataset, the optimal threshold of the created binary classifier with pre-selection used to classify a frame as containing a polyp was determined by using a ROC curve. To limit the number of frames that need to be manually re-assessed by an expert, a desired level of 90% specificity was used. 

The other three studies on CCE polyp detection developed a convolutional neural network (CNN) that classified frames as either “normal” or “containing a polyp/colorectal neoplasia/protruding lesion” [19,20,21]. CNN uses many images to develop and train a model by learning rich features from these images. Ideally, a large amount of data is needed to develop and train these models. However, available data in the form of CCE images is limited which makes it difficult to create a CNN for CCE polyp detection from scratch. To partially overcome this problem, all three studies used an existing CNN architecture and trained this model with CCE images to improve its performance. To test the performance of the proposed CNN models, all studies used separate images that were not used for the training of the models. The third study used manual analyses performed by trained nurses and gastro-enterologists as the reference group [19]. The fourth study used manual analyses performed by three expert gastroenterologists [20]. The fifth study used manual analyses performed by two expert gastroenterologists [21]. The proposed CNN model in the fourth study was not only developed to detect polyps but also colorectal cancer (colorectal neoplasia) and the CNN model in the fifth study was developed to detect protruding lesions such as polyps, epithelial tumors, submucosal tumors and nodes. These last two studies created a ROC curve to measure the performance of their CNN model.

#### 3.5.2. Performance of the Proposed AI models for Polyp Detection

The first study did not evaluate the accuracy of their developed algorithm in terms of sensitivity and specificity [17]. They only provided a description of the amount of protrusion into the lumen of CCE images expressed in *p*-values for different colonic anomalies. 80% of all polyps had a *p*-value higher than 500. All polyps that expressed a *p*-value higher than 2000 were polyps that were larger than 1 cm. The *p*-value was always under 500 in frames with cecal ulcer, diverticula, bubbles or trash liquid. However, some examples were shown that some folds mimicked polyps and were associated with a high *p*-value. 

The other studies did provide numbers on the accuracy of their AI models for automated polyp detection in CCE. The binary classifier with pre-selection developed in the second study resulted in a sensitivity of 47.4% and a specificity of 90.2% on a per frame basis using a threshold value of 37 [15]. Since in a clinical setting it is important that each polyp is detected in at least 1 frame, a ROC curve was also determined on a per polyp basis. At the same threshold value, this resulted in a sensitivity of 81.3% and a specificity of 90.2%. At a threshold of 40 a specificity of 93.5% was reached while maintaining the same per polyp sensitivity. 

Even though the CNN model created in the third study was only evaluated on a per frame basis, their model resulted in an even better performance with a sensitivity of 98.1% and a specificity of 96.3% [19]. 

The fourth study also evaluated performance on both per frame and per lesion basis, but again this did not result in a better performance than the CNN model in the third study. The model from the fourth study resulted in a sensitivity of 79.0% and a specificity of 87.0% for colorectal neoplasia on a per frame basis. Per lesion analysis increased the sensitivity to 96.2% [20]. The CNN model in the fifth study was only evaluated on a per frame basis and resulted in a sensitivity of 90.7% and a specificity of 92.6% [21].

### 3.6. Other Artificial Intelligence for CCE-2

Besides the studies on artificial intelligence for the assessment of bowel cleansing and polyp detection in CCE-2, two other studies were included. One study reported on the detection of blood in the colonic lumen [22]. They developed a convolutional neural network (CNN) that classified frames as either “normal” or “containing blood.” The same strategy for CNN development was used as in the previously mentioned study on polyp detection conducted by the same research group [21]. The CNN model only evaluated the presence of blood on a per frame basis and resulted in a sensitivity of 99.8% and a specificity of 93.2%. 

Another study reported on artificial intelligence for the localization of CCE-2 [18]. A model describing the shape of the intestine was created and feature points such as edges, corners, blobs or ridges were identified. Subsequently, capsule movement and speed were estimated by determining movement towards, away or rotated from these feature points, also taken the capsule’s frames per second (Hz) into account. The model was run many times and resulted in similar colonic shaped paths. Points usually associated with the ascending colon, hepatic flexure, transverse colon, splenic flexure and descending colon were identified. The model’s predictions of colonic sections were compared to expert labeled sections. The average accuracy of the model for frame colonic section classification was 86%.

## 4. Discussion

To our knowledge, this is the first systematic review providing an overview on the use of AI methods for reviewing CCE-2 colonic images. CCE provides a non-invasive alternative to colonoscopy for exploration of the colonic mucosa, but its applicability is limited by the accompanying labor-intensive reviewing time and by the risk of inter-observer variability. Automated reviewing of CCE images is an important step in the evolution of CCE. AI methods show promising results, with high sensitivity but lower specificity for the assessment of bowel cleansing and high sensitivity and specificity for polyp or colorectal neoplasia detection and blood detection.

Only one study reported on the AI assessment of CCE-2 bowel cleansing in terms of sensitivity and specificity [14]. However, this study shows promising results for its two developed CAC scores yielding high sensitivities (86.5% and 95.5% respectively) but lower specificities (78.2% and 63.0% respectively) for discriminating adequately cleansed from inadequately cleansed images. Adequately cleansed frames were only observed in 16.7% and 9.9%, respectively. CCE videos were excluded when they were identified as being too poor in quality after the first lecture and when they were incomplete, so the actual overall adequate cleansing levels were even lower. In a previous meta-analysis on the accuracy of CCE compared to colonoscopy, the rate of adequate bowel preparation varied from 40–100%, where most studies reported adequate cleansing levels over 80% [4]. The low number of adequately cleansed frames in the study included in this current review makes the risk of falsely identifying frames as adequately cleansed higher, which could explain the lower specificities of the CAC scores compared to its sensitivities. Since this was the only study reporting on AI for CCE bowel cleansing assessment in terms of sensitivity and specificity, the observed accuracy of bowel cleansing assessment by the CAC scores in this study cannot be compared to previous literature. However, optimal cut-off values yielding the highest diagnostic performances were determined for scores using a ROC curve, which could have led to overoptimistic results, which could likely be poorer when using the same threshold in an independent sample [13]. 

The other study reporting on the AI assessment of CCE bowel cleansing did not report accuracy results of their proposed AI models in terms of sensitivity and specificity or the percentage of adequately cleansed videos [16]. However, the low agreement levels of the non-linear index model (32%) and the SVM model (47%) with the reference group CCE readers are alarming. More studies on the AI assessment of CCE bowel cleansing in terms of sensitivity of specificity, with realistic adequate cleansing levels, are needed to be able to evaluate newly developed AI models accurately. 

The proposed AI models for polyp or colorectal neoplasia detection resulted in high sensitivities of 47.4–98.1% and high specificities of 87.0% to 96.3% in per-frame analysis [15,19,20,21]. Two studies performed per-lesion analysis, in addition to per-frame analysis, which improved sensitivity of polyp- or colorectal neoplasia detection to 81.3–98.1% [15,20]. It should be noted that the abovementioned results from four included AI studies were all compared to CCE-2 readers, so the concluded sensitivities and specificities represent the ability of the AI models to reach the same performance levels as CCE-2 readers. The previously mentioned meta-analysis on the accuracy of per-lesion detection by CCE-2 readers compared to colonoscopy reported a sensitivity of 85% and a specificity of 85% for polyps of any size [4]. 

One study determined the optimal threshold of their binary classifier with pre-selection by using a ROC curve, which may have led to overoptimistic estimates of its performance [15]. Still, the highest sensitivities were reached in the other three studies that developed a CNN model for polyp or colorectal neoplasia detection [19,20,21]. We believe future development of AI methods for reviewing CCE images should be focused on the creation of CNN models. While other AI methods fail to reach the same performance as humans, previous literature has shown that CNN is able to match human performance in different tasks [8,23]. However, optimal CNN requires training the algorithms with large amounts of data, which can be a challenge in the field of CCE for which the availability of data is limited. 

Only one study reported on the computed detection of blood in the colonic lumen [22]. Even so, their CNN model shows a promising result with a high sensitivity of 99.8%. Computed localization of the capsule within the colon was also only reported in one study. The accuracy for classifying frames to a specific colonic section was high (86%), but further studies are needed to validate this application in terms of sensitivity and specificity. 

While conducting our literature search, it was remarkable how many articles did not specify whether they used small bowel capsule endoscopy (SB-CE) or colon capsule endoscopy (CCE). Even when the use of CCE was reported, it was not always reported whether the first-generation (CCE-1) or second-generation (CCE-2) capsule was used. CCE-1 is an outdated version of the colon capsule with low sensitivity for detection of polyps compared to CCE-2. Therefore, articles not specifying the use of CCE-2 were excluded from this review. Future studies on the AI assessment of reviewing CCE images should report on the type of capsule that was used. 

Overall, literature on AI for reviewing CCE-2 colonic images is scarce. Two studies reported on the AI assessment of bowel cleansing and five studies reported on AI polyp or colorectal neoplasia detection. Only one study reported on the detection of blood in the colonic lumen and only one study created a rough AI model for determining the location of the capsule within the colon. The AI methods and study designs used were heterogeneous. Therefore, we could not perform a formal meta-analysis. Most studies had a limited sample size to test the performance of their AI models. Especially for studies using machine or deep learning, a large proportion of CCE images is needed for training the model, limiting the amount of images left for testing the models. Three out of nine studies included in this review did not report on the performance of their AI models in terms of sensitivity and specificity, making it hard to determine their value [16,17,18]. 

Nevertheless, the studies presented in this systematic review show promising results for the use of AI for reviewing CCE-2 colonic images with high sensitivities for both bowel cleansing assessment as well as polyp or colorectal neoplasia detection and blood detection. Manual CCE review is time-consuming and faces problems regarding inter observer variability. Improvements in imaging recognition will improve the reading time and inter observer variability, and may accelerate the use of CCE. This systematic review gives hope that AI can provide a timesaving, objective and reproducible method for reviewing CCE images.

### Necessary Action Points to Reach Implementation of AI Technology for CCE in Daily Practice

Actual implementation of AI for reviewing CCE-2 colonic images is a crucial step in the applicability of CCE in daily clinical practice. Future studies should preferably focus on CNN, because of its high potential for reaching human-like performance. In order to reach its implementation, several steps need to be taken. CNN algorithms need to be optimized and tested with more data, possibly requiring the set-up of a large international CCE database. To ensure adequate evaluation of the added value of the AI method, studies should always report the version of the capsule used and the accuracy of their models in terms of sensitivity and specificity. Additionally, studies should preferably only use the results from expert CCE readers to test the performance of their AI methods, since the concluded sensitivities and specificities represent the ability of the AI models to reach the same performance levels as these readers. Besides CNN, which requires an adequate number of coloscopy images, synthetic samples can also be used as artificial intelligence methods [24,25]. Finally, when these gaps and barriers have been overcome, prospective clinical trials have to confirm the accuracy of the optimized CNN models. 

## Figures and Tables

**Table 1:** Systematic literature search. * = *symbol that broadens a search by finding words that start with the same letters*.

**Embase.com (1971-)**
(‘capsule endoscopy’/exp OR ‘capsule endoscope’/de OR ((capsule * OR videocapsule *) NEAR/3 (endoscop * OR colonoscop *)):ab,ti) AND (‘large intestine’/exp OR ‘large intestine disease’/exp OR ‘large intestine tumor’/exp OR colonoscopy/exp OR (colon * OR colorectal * OR rectal OR rectum OR large-intestin *):ab,ti) AND (‘artificial intelligence’/exp OR ‘machine learning’/exp OR ‘software’/exp OR ‘algorithm’/exp OR automation/de OR ‘computer analysis’/de OR ‘computer assisted diagnosis’/de OR ‘image processing’/de OR ((artificial * NEAR/3 intelligen *) OR (machine NEAR/3 learning) OR (compute * NEAR/3 (aided OR assist * OR technique *)) OR software * OR algorithm * OR automat * OR (image NEAR/3 (processing OR matching OR analy *)) OR support-vector * OR svm OR hybrid * OR neural-network * OR autonom * OR (unsupervis * NEAR/3 (learn * OR classif *))):Ab,ti) NOT ([animals]/lim NOT [humans]/lim)
**Medline ALL Ovid (1946-)**
(Capsule Endoscopy/OR Capsule Endoscopes/OR ((capsule * OR videocapsule *) ADJ3 (endoscop * OR colonoscop *)).ab,ti.) AND (Intestine, Large/OR Colorectal Neoplasms/OR exp Colonoscopy/OR (colon * OR colorectal * OR rectal OR rectum OR large-intestin *).ab,ti.) AND (exp Artificial Intelligence/OR exp Machine Learning/OR Software/OR Algorithms/OR Automation/OR Diagnosis, Computer-Assisted/OR Image Processing, Computer-Assisted/OR ((artificial * ADJ3 intelligen *) OR (machine ADJ3 learning) OR (compute * ADJ3 (aided OR assist * OR technique *)) OR software * OR algorithm * OR automat * OR (image ADJ3 (processing OR matching OR analy *)) OR support-vector * OR svm OR hybrid * OR neural-network * OR autonom * OR (unsupervis * ADJ3 (learn * OR classif *))).ab,ti.) NOT (exp animals/ NOT humans/)
**Web of Science Core Collection (1975-)**
TS=((((capsule * OR videocapsule *) NEAR/2 (endoscop * OR colonoscop *))) AND ((colon * OR colorectal * OR rectal OR rectum OR large-intestin *)) AND (((artificial * NEAR/2 intelligen *) OR (machine NEAR/2 learning) OR (compute * NEAR/2 (aided OR assist * OR technique *)) OR software * OR algorithm * OR automat * OR (image NEAR/2 (processing OR matching OR analy *)) OR support-vector * OR svm OR hybrid * OR neural-network * OR autonom * OR (unsupervis * NEAR/2 (learn * OR classif *)))))
**Cochrane CENTRAL register of Trials (1992-)**
(((capsule * OR videocapsule *) NEAR/3 (endoscop * OR colonoscop *)):ab,ti) AND ((colon * OR colorectal * OR rectal OR rectum OR large-intestin *):ab,ti) AND (((artificial * NEAR/3 intelligen *) OR (machine NEAR/3 learning) OR (compute * NEAR/3 (aided OR assist * OR technique *)) OR software * OR algorithm * OR automat * OR (image NEAR/3 (processing OR matching OR analy *)) OR support-vector * OR svm OR hybrid * OR neural-network * OR autonom * OR (unsupervis * NEAR/3 (learn * OR classif *))):Ab,ti)
**Google scholar**
“capsule|videocapsule endoscopy|colonoscopy” colon|colonoscopy|colorectal “artificial intelligence”|”machine learning”|”computer aided|assisted”|software|algorithm|automated|”image processing|matching|analysis”|”support vector”|”neural network”

**Table 4:** Results of the two included studies examining computed assessment of bowel cleansing in CCE.

Study	Type of AI	Frames/Videos Analyzed, *n*	Adequately Cleansed Frames/Videos, %	Sensitivity, %	Specificity, %	PPV, %	NPV, %	Level of Agreement AI with Readers, %	Videos Misclassified More than One Class
Becq * [14]	R/G ratio	216 frames	16.7%	86.5%	78.2%	45.1%	96.6%	-	-
	R/(R + G) ratio	192 frames	9.9%	95.5%	63.0%	25.0%	99.0%	-	-
Buijs ** [16]	Non-linear index model	41 videos	Unknown	-	-	-	-	32%	32%
	SVM model	41 videos	Unknown	-	-	-	-	47%	12%

AI = Artificial Intelligence, PPV = Positive Predictive Value, NPV = Negative Predictive Value, R/G = Red over Green, R/(R+G) = Red over Brown, SVM = Support Vector Machine, CCE = Colon Capsule Endoscopy. The computed assessment of cleansing (CAC) scores developed by Becq et al. resulted in a bowel cleansing evaluation for each frame defined as either adequately or inadequately cleansed. The CAC models developed by Buijs et al. resulted in a bowel cleansing classification for each video defined as either unacceptable, poor, fair or good. * The percentage of adequately cleansed frames/videos was based on the evaluation by the reference group. ** 31 adequately cleansed (fair or good) and 10 inadequately cleansed (unacceptable or poor) videos were selected from a previous trial. The videos were re-evaluated by the reference group in this study, however, numbers on the adequate cleansing levels from these evaluations were not reported.

**Table 5:** Results of the five included studies examining computed polyp- or colorectal neoplasia detection in CCE.

Study	Type of AI	Application	Frames Analyzed, *n*	Amount of Polyps or Colorectal Neoplasia, *n*	Amount of Frames Containing Polyps, *n*	Cut-off Value	Accuracy	Sensitivity on a per Frame Basis, %	Specificity on a per Frame Basis, %	Sensitivity on a per Polyp Basis, %	Specificity on a per Polyp Basis, %
Figueiredo [17]	Protrusion based algorithm	Polyp detection	1700	10	Unknown	-	-	-	-	-	-
Mamonov [15]	Binary classification after pre-selection	Polyp detection	18,968	16	230	37	-	47.4%	90.2%	81.3%	90.2%
						40	-	-	-	81.3%	93.5%
Nadimi * [19]	CNN	Polyp detection	1695	Unknown	Unknown	-	98.0%	98.1%	96.3%	-	-
Yamada ** [20]	CNN	Colorectal neoplasia detection	4784	105	Unknown	-	83.9%	79.0%	87.0%	96.2%	Unknown
Saraiva [21]	CNN	Protruding lesion detection	728	Unknown	172	-	92.2%	90.7%	92.6%	-	-

AI = Artificial Intelligence, CNN = Convolutional Neural Network. Unknown means the numbers were not described, - means the numbers were not part of the outcomes of the study. * The entire dataset consisted of 11,300 CCE images of which 4800 contained colorectal polyps. Of the entire dataset, 15% was used to test the performance of the CNN. The amount of frames containing a polyp in this test dataset was not described. ** From the 105 observed colorectal neoplasia, 103 were polyps and 2 were colorectal cancers. 1850 images of patients with colorectal neoplasia were included. It was not described how many of the frames of the CCE-2 videos of these patients contained polyps or colorectal cancers.

## Data Availability

No new data were created or analyzed in this study. Data sharing is not applicable to this article.

## Abbreviations

CCE = Colon Capsule Endoscopy; GI = Gastrointestinal; ESGE = European Society of Gastrointestinal Endoscopy; FDA = Food and Drug Administration; AI = Artificial Intelligence; SVM = Support Vector Machine; CNN = Convolutional Neural Network.
